# Acetylcholinesterases from the Disease Vectors *Aedes aegypti* and *Anopheles gambiae*: Functional Characterization and Comparisons with Vertebrate Orthologues

**DOI:** 10.1371/journal.pone.0138598

**Published:** 2015-10-08

**Authors:** Cecilia Engdahl, Sofie Knutsson, Sten-Åke Fredriksson, Anna Linusson, Göran Bucht, Fredrik Ekström

**Affiliations:** 1 Department of Chemistry, Umeå University, Umeå, Sweden; 2 Swedish Defence Research Agency, CBRN Defence and Security, Umeå, Sweden; Weizmann Institute of Science, ISRAEL

## Abstract

Mosquitoes of the *Anopheles (An*.*)* and *Aedes (Ae*.*)* genus are principal vectors of human diseases including malaria, dengue and yellow fever. Insecticide-based vector control is an established and important way of preventing transmission of such infections. Currently used insecticides can efficiently control mosquito populations, but there are growing concerns about emerging resistance, off-target toxicity and their ability to alter ecosystems. A potential target for the development of insecticides with reduced off-target toxicity is the cholinergic enzyme acetylcholinesterase (AChE). Herein, we report cloning, baculoviral expression and functional characterization of the wild-type AChE genes (*ace-1)* from *An*. *gambiae* and *Ae*. *aegypti*, including a naturally occurring insecticide-resistant (G119S) mutant of *An*. *gambiae*. Using enzymatic digestion and liquid chromatography-tandem mass spectrometry we found that the secreted proteins were post-translationally modified. The Michaelis-Menten constants and turnover numbers of the mosquito enzymes were lower than those of the orthologous AChEs from *Mus musculus* and *Homo sapiens*. We also found that the G119S substitution reduced the turnover rate of substrates and the potency of selected covalent inhibitors. Furthermore, non-covalent inhibitors were less sensitive to the G119S substitution and differentiate the mosquito enzymes from corresponding vertebrate enzymes. Our findings indicate that it may be possible to develop selective non-covalent inhibitors that effectively target both the wild-type and insecticide resistant mutants of mosquito AChE.

## Introduction

Mosquitoes are estimated to transmit infectious diseases to more than 500 million people in Africa, Americas and Asia, with millions of deaths annually. Climate change, globalization and other factors indicate that there is a growing risk that these infectious diseases may also become a problem in Europe [1]. Malaria and dengue are the most common mosquito-borne infectious diseases, each responsible for millions of cases per year. The uncertainty in the number of reported cases is large with approximately 200 million cases of malaria in 2013, along with more than half a million deaths due to the disease [2]. In addition there were almost 100 million cases of dengue infections in 2010 [3]. The responsible agents of these diseases are transmitted through the bites of infected mosquitoes. Species of the *Anopheles* genus transmit the malaria parasite, with *Anopheles (An*.*) gambiae* as the main vector [4]. Similarly, *Aedes (Ae*.*)* mosquitoes such as *Ae*. *aegypti* and *Ae*. *albopictus* are the principal vectors of dengue as well as yellow fever and chikungunya viruses [5, 6].

Reducing the numbers of disease-transmitting mosquitoes is a proven strategy for controlling disease transmission. The four main classes of insecticides used for mosquito vector control are chlorinated hydrocarbons, organophosphates, carbamates and pyrethroids [7]. Although their molecular targets differ, these insecticides have similar effects on their target organisms, leading to paralysis and death. Chlorinated hydrocarbons, such as dichlorodiphenyltrichloroethane (DDT), and pyrethroids target the voltage-gated ion channels of neurons, while organophosphates and carbamates inhibit the activity of acetylcholinesterase (AChE, EC 3.1.1.7), an essential enzyme for the function of the nervous system. The large-scale production and frequent use of insecticides has caused their accumulation in ecosystems, resulting in environmental contamination and toxicity to many different species including humans [7]. The spread of insecticide resistance also threatens the effectiveness of currently used insecticides [8–10]. In particular, there have been alarming reports from 27 countries in sub-Saharan Africa of pyrethroid resistance among *Anopheles* vectors [11]. New vector control strategies such as the use of microorganisms, viruses, biological toxins or natural products, collectively called biopesticides, are under development [12–14]. However, to maximize the effectiveness of vector control and combat the spread of mosquito-borne diseases, it may be useful to adopt combinations of different approaches [15]

**Fig 1:**
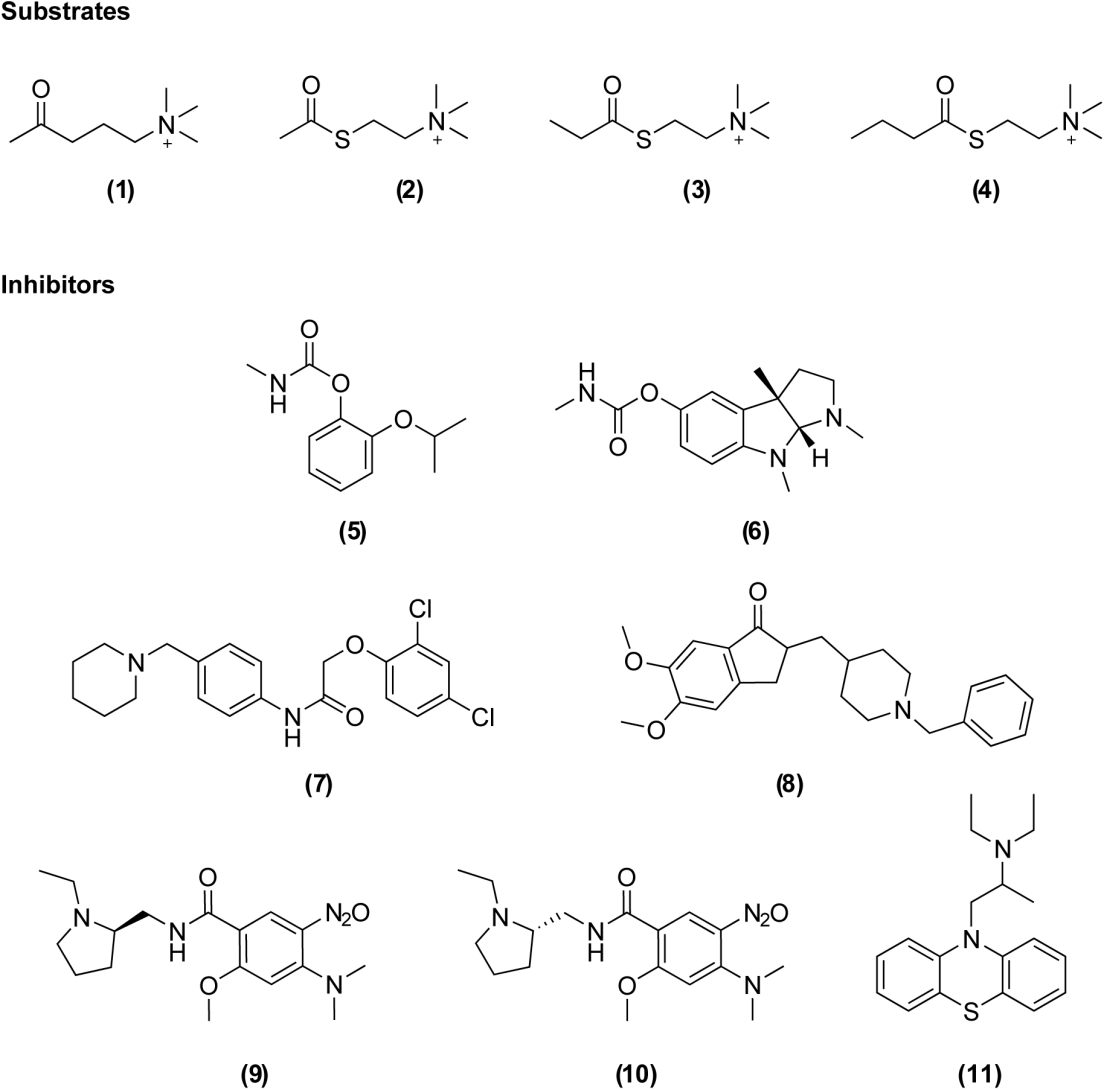
Chemical structures of the substrates and inhibitors investigated in this study. Substrates: acetylcholine (**1**), acetylthiocholine (**2**), propionylthiocholine (**3**), butyrylthiocholine (**4**). Covalent inhibitors: propoxur (**5**), eserine (**6**). Non-covalent inhibitors: C7653 (**7**), donepezil (**8**), C5681R (**9**), C5681S (**10**), ethopropazine (**11**).

AChE is not only a target for insecticides but also for chemically related warfare agents (*i*.*e*. nerve agents) and naturally occurring toxins (*e*.*g*. snake venoms). It is responsible for terminating nerve signals at the synaptic cleft by hydrolyzing the neurotransmitter acetylcholine (ACh (**1**), Fig 1). Disruption of this mechanism leads to accumulation of ACh causing overstimulation and eventual blockage of neurotransmission. Currently used anticholinesterase insecticides for vector control are inhibitors that form a covalent bond with a conserved serine at the active site of the enzyme (reviewed in [16]). This active site serine (Ser^203^, human AChE numbering) is located at the base of a deep and narrow gorge, where it forms one part of the catalytic triad together with His^447^ and Glu^334^ [17, 18]. The gorge is lined with aromatic residues and penetrates halfway through the enzyme.

Mammals have one *ace* gene encoding for AChE and this is also the case for *Drosophila (D*.*) melanogaster* [19]. However, it is now established that most investigated insects carry multiple AChE genes. Mosquitoes have two AChE genes due to an old duplication; the paralogous *ace-1* gene and the orthologous *ace-2* gene which is homologous to the gene in *Drosophila* [20–22]. *D*. *melanogaster*, and other flies, possess only one gene, probably due to a secondary loss during the evolution of the Diptera [23]. It is AChE1, encoded by the *ace-1* gene, which is responsible for catalytic acetylcholinesterase activity and AChE-mediated insecticide resistance in mosquitoes [21, 24]. A naturally occurring mutation that confers resistance to organophosphates and carbamates in mosquitoes is a glycine to serine mutation at position 119 (G119S) in AChE1 [24]. This mutation has been identified in populations of *An*. *gambiae*, as well as in the West Nile and Japanese encephalitis vector *Culex pipiens*, found in different geographical locations [24]. However, this mutation has not been found in *Ae*. *aegypti ace-1*, probably because this gene uses a different codon for glycine at this position, necessitating two point mutations for the conversion of glycine to serine [25].

The crystal structure of AChE from *D*. *melanogaster* has been determined [26], but no structure of mosquito AChE is currently available. AChE from *D*. *melanogaster* shows an amino acid sequence identity of 39 and 37% to the corresponding enzymes from *An*. *gambiae* and *Ae*. *aegypti*, respectively. This is comparable to the sequence identity of 38% between *ace-1* and *ace-2* within *An*. *gambiae*. Notably, the mosquito AChE1 and the human enzyme exhibit a slightly higher sequence identity (48–49%). Most studies on AChEs have focused on AChE from *Homo sapiens*, *Mus musculus*, *D*. *melanogaster* and the electric rays *Torpedo californica* or *marmorata*. Recently, AChE1 from *An*. *gambiae* (*Ag*AChE1), and to some extent, *Ae*. *aegypti* (*Aa*AChE1), has been expressed and biochemically characterized [27–29]. In addition, unique residues in the active site of AChE1 have been identified and suggested as targets for insecticides [30, 31]. It has also been shown that some covalent inhibitors targeting *Ag*AChE1 show selectivity over human AChE [32].

Here we report the cloning, expression and profiling of catalytically active wild-type enzymes encoded by the *ace-1* genes of *An*. *gambiae* and *Ae*. *aegypti* as well as the G119S mutant of *An*. *gambiae*. Post-translational modifications of these enzymes were investigated using LC-MS/MS and their basic catalytic parameters and substrate preferences were determined. Furthermore, ligand-binding properties were investigated using a set of inhibitors (**5–11**, Fig 1) and compared to those of vertebrate AChEs from *Homo sapiens* (*h*AChE) and *Mus musculus* (*m*AChE).

## Methods

### Construction of synthetic genes and cloning into the baculovirus chromosome

Full-length sequences of AChE1 (*ace-1*) from *An*. *gambiae* (XM_321792) [33] and *Ae*. *aegypti* (EF209048) [28] were downloaded from GenBank and codon-optimized for expression in *Spodoptera frugiperda*-9 (*Sf* 9) cells (ATCC^®^ CRL-1711™) according to the codon database [34]. Synthetic genes covering the complete open-reading frames were produced (Eurofins MWG Operon, Germany) and cloned in frame to a C-terminal 6xHis-tag in the baculovirus donor vector pFastBac/CT-TOPO (Invitrogen, Waltham, MA, USA). Correct sequences of individual clones were verified by sequencing plasmid DNA from One Shot Mach1-T1^R^ *E*. *coli*. The *Ag*AChE1-G119S mutant was constructed using the Quick-Change II XL Site Directed Mutagenesis kit (Agilent Technologies, Santa Clara, CA, USA) and the mutation was confirmed by sequencing.

The donor plasmids carrying the *ace-1* genes were then transformed into MAX Efficiency DH10Bac competent *E*. *coli* carrying the bacmid chromosome along with a helper plasmid that enables recombination. The genes of interest were recombined into the baculovirus chromosome according to the Bac-to-Bac TOPO Expression system manual (Invitrogen) and bacterial colonies carrying the *ace-1* genes from *An*. *gambiae* or *Ae*. *aegypti* were identified by blue/white screening before sequencing. Bacmid DNA with the expected sequence was gently isolated from the bacteria prior to transfection using Fugene^®^HD (Roche Applied Science, Penzberg, Germany) into *Sf*9 cells grown in Sf900 serum-free insect cell growth medium (Invitrogen).

### Expression

The baculovirus infections induced as described above were verified 48–72 h post-infection by GFP fluorescence in Sf9 Easy Titer (*Sf*9-ET) cells maintained in HyClone SFX Insect medium supplemented with geniticin (100 μg/ml) and FBS (5%) [35] and by measuring the AChE1 activity of supernatants of baculovirus-infected *Sf* 9 cells (see the section on “determination of enzymatic activity”). Viral titers in expanded viral stocks were determined at day five by end-point dilution assays in the *Sf* 9-ET cell line by scoring GFP-positive wells [36]. The expression of *ace-1* gene products was optimized by analysing AChE activity at multiple time-points after infection. Briefly, adherent *Sf*9 cells at approximately 70-80% confluence were infected with a multiplicity of infection (MOI), *i*.*e*. ratio of virus to insect cells, of 0.1, 1.0 and 10. Infected cells were thereafter maintained for 48 h at 28°C before the media was replaced and the cells were incubated for an additional 3–7 days at 20°C or 28°C. Cells were harvested by scraping and separated from the media by centrifugation for 10 min at 500 rcf. The enzymatic activity was determined as described below in samples of infected cells and in corresponding cell culture media using a PerkinElmer Lambda 650 UV/VIS spectrometer at 412 nm. Once an optimal MOI, temperature and duration of infection had been determined, the production of AChE1 was scaled up. AChE1 proteins were expressed from full-length genes and not truncated prior to cloning, these recombinant proteins were used for all experiments**.** The cloning and expression of *h*AChE and *m*AChE have been described previously [37, 38].

### Purification

The expressed wild-type proteins were purified by affinity chromatography using the active site ligand procainamide coupled to epoxy-activated sepharose (GE healthcare LifeScience, Little Chalfont, UK) [38]. Cell culture supernatants were mixed with procainamide sepharose and the slurry was incubated for 16 h at 4°C under constant rotation. It was then loaded on Eco columns (Bio-Rad Laboratories Inc., Hercules, CA, USA), washed with 2 mM MES (pH 6.5) containing 250 mM NaCl and finally eluted with the same buffer supplemented with 50 mM procainamide. Attempts to use the C-terminal His-tag for Ni-NTA agarose purification were not successful. The purified samples were analysed by SDS-PAGE and subsequently used for the analysis of post-translational modifications and for *k*_*cat*_ calculations. Non-purified secreted enzymes were used for all other experiments.

### Glycosylation analysis

The glycosylation of the expressed recombinant AChE1s was investigated using enzymatic digestion and liquid chromatography-tandem mass spectrometry (LC-MS/MS). The purified protein was digested in a molecular weight cut-off centrifugal filter (Microcon 10,000 MWCO, Millipore, Billerica, MA, USA) [39]. A 100 μl aliquot was concentrated on the filter at 12,000 x g, after which the filter was washed twice with 200 μl ammonium bicarbonate (100 mM). The protein was subsequently digested with 1 μg of proteinase K in 100 μl of 100 mM ammonium bicarbonate for 4h at 40°C and the peptides were recovered by centrifugation and washing of the filter with 100 μl of 50% acetonitrile. The digests were stored at -20°C before LC-MS analysis.

The digested proteins were analysed by LC-MS and MS/MS on a Waters Nano-Acquity UPLC system connected to a Waters Qtof Ultima mass spectrometer equipped with a nano-electrospray ion source (Waters Inc). The peptides were separated on a 100 mm, 75 μm i.d. C18 UPLC column (Waters Inc.) using a water:acetonitrile gradient containing 0.1% formic acid from 3–40% acetonitrile over 25 min at a flow rate of 400 nl/min. The samples were analysed by LC-MS using alternate scanning at low and elevated collision energies with argon as the collision target. Carbohydrate marker ions at *m/z* 204.1 and 366.2 formed by collision-induced dissociation (CID) at 40 eV were used to detect glycopeptides. The corresponding low energy mass spectrum was examined and the product ion spectra of selected precursor ions were acquired in a separate LC-MS/MS run.

### Activity assay and determination of enzymatic activity

The enzymatic activity was investigated using the Ellman assay [40]. The assay was performed using the substrate acetylthiocholine iodide (ATChI, **2**) at a concentration of 1 mM in 0.1 M sodium phosphate buffer (pH 7.4) at 30°C. The reagent 5,5'-dithiobis-(2-nitrobenzoic acid) (DTNB) was present at a final concentration of 0.2 mM. Enzyme-catalysed hydrolysis of the substrate was quantified by monitoring a time-dependent change in absorbance at 412 nm due to the colour change caused by the reaction between DTNB and the hydrolysis product. Enzymatic activities were evaluated by plotting absorption per minute (dA/min) versus substrate concentration ([S]). The substrates’ rates of autohydrolysis were subtracted from the measured enzyme-catalyzed rates. All experiments were performed at least in duplicate and values are given with a 95% confidence interval (CI).

### Michaelis-Menten kinetics

Michaelis-Menten constants (*K*_*m*_) and maximum velocity (*V*_*max*_) values for *Ag*AChE1, *Ag*AChE1-G119S, *Aa*AChE1, *m*AChE and *h*AChE were determined by measuring their initial rates (*V*_*0*_) at different substrate concentrations. Substrate concentrations where substrate inhibition was recognizable were avoided and the *K*_*m*_ and *V*_*max*_ values were obtained from non-linear regression curve fit using the Michaelis-Menten equation in GraphPad Prism version 6.04 for Windows (GraphPad Software, La Jolla, CA, USA, www.graphpad.com). The substrate preferences of *Ag*AChE1, *Ag*AChE1-G119S and *Aa*AChE1 were investigated using ATChI, propionylthiocholine iodide (PTChI) and butyrylthiocholine iodide (BTChI) (**2–4**, Fig 1). Each enzyme’s turnover number (*k*_*cat*_) was determined from the relationship between *V*_*max*_ and protein concentration. The protein concentration as the total number of active sites in solution at a given volume was determined experimentally by active site titrations using the covalent inhibitor O-ethyl S-[2-(diisopropylamino) ethyl] methylphosphonothioate (VX). The wild-type mosquito enzymes were incubated with different concentrations of VX for 120 min to achieve complete inactivation of the enzymes by both enantiomers of VX. Enzymatic activity was then measured and a titration curve was constructed by plotting the relative activity against the VX concentration. The number of active sites was determined from the titration and used together with *V*_*max*_ for the calculation of *k*_*cat*_.

### Inhibition kinetics

Inhibition profiles of different AChEs were determined by studying a number of previously described AChE inhibitors (**5–11**, Fig 1) [41–43]. The non-covalent inhibitors (**7–11**) were characterized by determination of their half-maximal inhibitory concentration (*IC*_*50*_) values and covalently binding inhibitors (**5–7)** were investigated by determination of their inhibition constants (*k*_*i*_). Stock solutions of the inhibitors were prepared in dimethyl sulfoxide (DMSO) at a concentration of 100 mM and working dilutions thereof were prepared in 0.1 M sodium phosphate buffer pH 7.4. For *IC*_*50*_ determinations, the enzymatic activity was determined immediately following addition of inhibitor solutions of different concentrations up to a maximum of 1 mM. *IC*_*50*_ *-*values were calculated using non-linear regression (curve fitting) in GraphPad Prism and the log (inhibitor) vs. response variable slope equation with four parameters. For *k*_*i*_-determinations, samples were pre-incubated in the presence of the relevant inhibitor at the specified concentration and activity was measured at several time-points (1, 3, 5, 10, 20, 30, 40, 50 and 60 min) until no further decrease in activity could be seen. *k*_*obs*_ values were obtained using non-linear regression (curve fitting) in GraphPad Prism software with the one phase decay equation. These values were plotted as a function of the inhibitor concentration and the value of *k*_*i*_ was obtained from the resulting slope.

### Cluster analysis based on kinetic data

The relationships between different AChEs (*Aa*AChE1, *Ag*AChE1, *Ag*AChE1-G119S, *h*AChE and *m*AChE) with respect to their kinetic parameters were visualized by cluster analysis. The analysis was based on our experimental kinetic measurements together with previously published data, and its results were presented in the form of un-rooted trees.

Three cluster analyses of the AChEs were performed based on all of the parameters considered in this work and two different sub-sets of these parameters. Sub-set A contains parameters related to basal enzymatic function that are dependent on a reaction with the catalytic serine residue (*V*_*max*_, *K*_*M*_, *k*_*cat*_ and *k*_*i*_) while sub-set B contains parameters related to affinity for non-covalent inhibitors alone (*IC*_*50*_). Prior to the cluster analysis, the parameter values were scaled to unit variance and the *V*_*max*_ values of the investigated substrates of each enzyme were normalized against that enzyme’s *V*_*max*_ for ATChI. Euclidean distances between the enzymes were calculated for each of the three datasets. The enzymes were clustered according to these Euclidean distances and the Neighbor-Joining method [44] with a randomized order (seed 5) of the enzymes using PHYLIP [45]. The clustering is visualized as un-rooted trees, with the angle of the arc set to 360 degrees, where the branch lengths correspond to the Euclidean distances between the enzymes, reflecting the similarities and dissimilarities in the underlying data.

## Results and Discussion

### Design and cloning of expression constructs

To facilitate post-translational modifications such as translocation and cleavage of a putative secretion signal, an *Sf* 9 based baculovirus expression system was evaluated. Codon-optimized full-length *ace-1* genes of *An*. *gambiae* and *Ae*. *aegypti* were produced and cloned into donor plasmids. All plasmids containing the *ace-1* genes were verified by sequencing before being recombined into bacmid chromosomes. Recombinant baculovirus chromosomes were identified by blue/white screening and the flanking sequences of the inserts were reconfirmed by sequencing. Bacmid chromosomes were finally isolated and used for transfection into *Sf* 9 cell lines. Cells with typical morphological changes and granular appearance were noticed within one week of infection in *Sf* 9 cells, and a clear GFP fluorescence signal was subsequently observed in *Sf* 9-ET cells. Infected primary cultures (P1) were harvested and used for reinfection of *Sf* 9 cells before viral titers were determined in amplified viral stocks. The median tissue culture infective doses (TCID_50_) of the P2 stocks were calculated to be 2 x 10^8^ and 4 x 10^8^ / ml for *An*. *gambiae* and *Ae*. *aegypti*, respectively. The G119S mutant was made by site-directed mutagenesis of the wild type *An*. *gambiae ace-1* gene in the baculovirus donor vector before being recombined onto a baculovirus chromosome and expressed in *Sf* 9 cells like the wild type enzymes.

### Optimization of protein expression

The production and secretion of AChE1 was optimized by exploring the influence of incubation temperature, MOI and time of cultivation. The amount of expressed and secreted AChE1 was determined in the culture media and the highest secretion levels were obtained when adherent cells were infected at an approximate cell density of 70–80% confluence with a MOI of 0.1. The amount of secreted AChE1 at 5–7 days post-infection was estimated to be around 10 mg/L at 28°C, and slightly higher levels of expression were detected in suspension cultures. Of the total measured enzyme activity, about thirty percent was detected in cell culture media and the remaining enzyme activity was detected in samples of lysed cells. Under comparable expression conditions, media containing the *Ag*AChE1-G119S mutant enzyme displayed a significantly lower enzymatic activity than media collected from cells expressing the corresponding wild type enzyme.

### Characterization of AChE1 from mosquitoes

SDS-PAGE gel electrophoresis of the purified mature proteins showed that the secreted proteins migrated with an apparent molecular weight between 60–70 kDa (S1 Fig). Assuming that the mature proteins start at the amino acids D^162^NDP in the case of *Ag*AChE1 [27] and D^129^NDP in that of *Aa*AChE1 [28], their theoretical weight is 64 kDa. To assess the glycosylation of the recombinant enzymes, purified mature proteins were studied after digestion with Proteinase K followed by LC-MS/MS analysis. Carbohydrate marker ions in the high energy spectrum indicated the presence of glycopeptides (S2 Fig) [46]. Further analysis (see also supporting information section S1 Text) revealed that the mass spectral data corresponded to a N-glycosylated core structure -GlcNAc(Fuc)-GlcNAc-Man_3_ (Table 1). In addition to the core structure, two additional glycoforms of the peptide containing terminal galactosamine were identified (Table 1). The structures were common to both species and consistent with the biantennary complex-type structures reported previously in fetal bovine serum AChE and equine serum BuChE [47]. To identify the specific glycosylation site, the peptide mass was matched to the amino acid sequence of the respective protein and we found that the glycan structures were attached to Asn^509^ in both species. Jiang *et al* have previously shown an increase in migration on SDS-PAGE for N-glycosidase-treated *Ag*AChE1 compared to non-treated samples, indicating the presence of N-glycosylated amino acids in *An*. *gambiae* [27].

**Table 1:** AChE1 glycopeptides detected by LC-MS.

Amino acid sequence and position	Corresponding glycan	Glycopeptide *m/z*	Glycopeptide mass	Glycan mass	Peptide mass
***Aedes aegypti***					
GLNTT 507–511	-GlcNAc (Fuc) GlcNAc Man (Man) Man	772.27^2+^	1542.52	1038.29	504.23
GLNTT 507–511	-GlcNAc (Fuc) GlcNAc Man (Man) Man GalNAc	873.79^2+^	1745.57	1241.34	504.23
GLNTT 507–511	-GlcNAc (Fuc) GlcNAc Man (Man GalNAc) Man GalNAc	975.32^2+^	1948.63	1444.40	504.23
*Anopheles gambiae*					
GLNTS 507–511	-GlcNAc (Fuc) GlcNAc Man (Man) Man	765.26^2+^	1528.51	1038.27	490.24
GLNTS 507–511	-GlcNAc (Fuc) GlcNAc Man (Man) Man GalNAc	866.80^2+^	1731.58	n. d.	n. d.
GLNTS 507–511	-GlcNAc (Fuc) GlcNAc Man (Man GalNAc) Man GalNAc	968.33^2+^	1934.65	n. d.	n. d.

n.d. = not determined

### Substrate preferences and substrate inhibition

**Fig 2:**
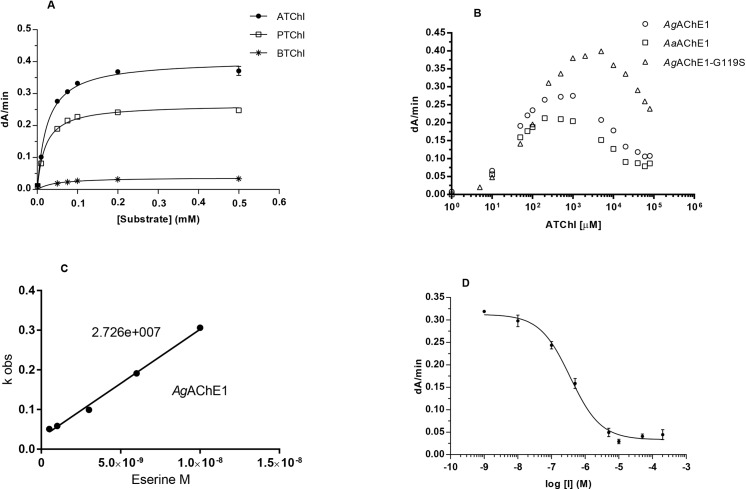
Enzyme kinetics. (A) Substrate preference for *Aa*AChE1 illustrated by Michaelis-Menten curves. The same trend was observed for *Ag*AChE1 *and Ag*AChE1-G119S. (B) Substrate inhibition of *Aa*AChE1, *Ag*AChE1 and *Ag*AChE1-G119S using ATChI as substrate. (C) Typical graph for *k*_*i*_-calculations of *Ag*AChE1 with eserine as inhibitor. (D) Typical dose-response curve of *Aa*AChE1 with inhibitor, used for determination of *IC*_*50*_-values for various compounds.

To investigate the substrate preferences of *Ag*AChE1, *Ag*AChE1-G119S and *Aa*AChE1, their Michaelis-Menten constants (*K*_*M*_) and maximum reaction rates (*V*_*max*_) were determined using the potential substrates ATChI (**2**), PTChI (**3**) and BTChI (**4**) (Figs 1 and 2A). For the wild-type enzymes, the *K*_*M*_ values were similar for all tested substrates, ranging from 24–36 μM for *Ag*AChE1 and 20–45 μM for *Aa*AChE1. Conversely, the *Ag*AChE1-G119S mutant exhibited a significant decrease in substrate affinity with increasing substrate size (MW_ATChI_ < MW_PTChI_ < MW_BTChI_); its *K*_*M*_ values were 58, 315 and 431 μM for ATChI, PTChI and BTChI respectively. For all enzymes, the *V*_*max*_ values decreased with increasing substrate size (ATChI < PTChI < BTChI) and were markedly lower for the hydrolysis of the largest substrate, BTChI (see Table 2). The relative catalytic efficiencies given by the *V*_*max*_ */K*_*M*_-ratios for the substrates ATChI, PTChI and BTChI were 19.7, 16.6 and 1.6 for *Ag*AChE1; 16.2, 13.2 and 0.8 for *Aa*AChE1; and 5.5, 0.27 and 0.08 for the G119S mutant, respectively. The AChE1 enzymes hydrolysed ATChI more efficiently than PTChI and BTChI, which is consistent with the behavior of *m*AChE and *h*AChE (Table 2) [48–50] and also with results previously obtained for various invertebrate AChE enzymes [27, 51, 52]. A common feature of vertebrate AChEs is that they show substrate inhibition at elevated substrate concentrations. Our analysis revealed that *Ag*AChE1, *Ag*AChE1-G119S and *Aa*AChE1 are also subject to such inhibition (Fig 2B), in agreement with a study on the biochemical profile of *Ag*AChE1 [27].

**Table 2:** Kinetic parameters of different AChEs.

Substrate	V_max_ (mA/min)	K_m_ (μM)	V_max_/K_M_	K_cat_ (s^-1^)
*Aedes aegypti *AChE1				
ATChI	406 (389–422)	25 (20–30)	16.2	140
PTChI	266 (258–275)	20 (17–23)	13.3	92
BTChI	38 (36–40)	45 (36–54)	0.8	13
*Anopheles gambiae* AChE1				
ATChI	528 (505–552)	27 (21–33)	19.6	124
PTChI	407 (393–421)	25 (20–29)	16.3	95
BTChI	58 (55–61)	36 (27–45)	1.6	14
*Anopheles gambiae* AChE1-G119S				
ATChI	318 (310–326)	58 (52–65)	5.5	n. d.
PTChI	105 (87–123)	303 (172–435)	0.27	n. d.
BTChI	34 (24–45)	431 (136–727)	0.08	n. d.
*Mus musculus* AChE				
ATChI	725 (694–756)	84 (72–95)	8.6	n. d.
PTChI	367 (354–380)	59 (51–66)	6.2	n. d.
*Homo sapiens* AChE				
ATChI	821 (782–860)	146 (128–165)	5.6	n. d.
PTChI	429 (415–442)	150 (137–162)	2.9	n. d.

n.d. = not determined, parentheses = 95% CI

### Michaelis-Menten kinetics

The kinetics was further investigated using the substrate analogue ATChI. The initial enzyme activities were monitored at different substrate concentrations and the *K*_*M*_ and *V*_*max*_ values were determined and compared to the corresponding constants for the vertebrate AChEs (Fig 2A and Table 2). The mosquito enzymes displayed a higher affinity for ATChI than the vertebrate AChEs. The *K*_*M*_ values for *Ag*AChE1, *Ag*AChE1-G119S and *Aa*AChE1 were 27, 58 and 25 μM, respectively, compared to 84 μM and 146 μM for *m*AChE and *h*AChE, respectively.

The kinetics of inhibition and the relationship between the protein concentration (*i*.*e*. the total number of active sites in solution) and enzymatic activity were investigated by means of a time-course experiment using the covalent inhibitor VX. The biphasic shape of the resulting curves indicates that both enantiomers of VX reacted (S3A Fig). For *Ag*AChE1 and *Aa*AChE1, the covalent modification progressed slowly and reached a plateau after approximately 120 minutes, allowing the construction of a titration plot (S3B Fig). From this titration, the relation between *V*_*max*_ and the total number of active sites in solution was established, allowing determination of the turnover number (*k*_*cat*_). The *k*_*cat*_ values were determined to be 124 s^-1^ and 140 s^-1^ for *Ag*AChE1 and *Aa*AChE1, respectively. These values are approximately twenty to forty times lower than those reported for vertebrate AChE [48, 53]. The *k*_*cat*_ values presented here are also lower than the previously reported values for *Ag*AChE1 (650 s^-1^ [27] and 3000 s^-1^ [54]); to our knowledge, no *k*_*cat*_ has been previously reported for *Aa*AChE1. The large differences between the reported values for *Ag*AChE1 may be due to different experimental methods. For example, the constants reported herein were determined using full length secreted enzyme and the concentration was determined using active site titrations. In contrast, previous reports use a colorimetric assay to determine the concentration of a purified, truncated construct. Because *Ag*AChE1-G119S was resistant towards the organophosphate VX (its activity decreased by less than 5% over 120 min of incubation) we were unable to perform similar titrations with this enzyme or to determine its *k*_*cat*_ value.

### Probing the active site gorge with inhibitors

The ligand binding properties of the AChEs were investigated by determining the potency of a set of known inhibitors including the covalent inhibitors propoxur (**5**) and eserine (**6**), and non-covalent inhibitors C7653 (**7**), donepezil (**8**), C5685R (**9**), C5685S (**10**) and ethopropazine (**11**) (Fig 1). Propoxur is used against insect pests for both agricultural and public health purposes [55], eserine and donepezil are used clinically in the treatment of neurological disorders [43], while ethopropazine has mainly been used as a biochemical tool to distinguish between cholinesterases and also for determining BuChE-specific tissues [50, 56]. The non-covalent inhibitory activity of C7653, C5685R and C5685S has been studied against both *h*AChE and *m*AChE [41, 42].

The covalent inhibitors propoxur and eserine were approximately 10-fold more potent inhibitors of AChE1 than of vertebrate AChEs, as shown by their inhibition constants (*k*_*i*_, Fig 2C and Table 3). The *k*_*i*_-values for eserine were 24.8 and 27.3 μM ^-1^ min^-1^ for the wild-type mosquito AChE1s and 1.4 and 2.7 μM ^-1^ min^-1^ for *m*AChE- and *h*AChE respectively. Interestingly, with a *k*_*i*_ of 1.18 μM ^-1^ min^-1^, the sensitivity of *Ag*AChE1-G119S was similar to that of *m*AChE and *h*AChE. The selectivity of propoxur followed the same general trend as eserine, but displayed 15–30 fold lower constants. Notably, the G119S mutant was very resistant to inhibition by propoxur and only marginal inhibition was observed (data not shown).

**Table 3:** Characterization of inhibitors.

*IC*_*50*_-values of reversible inhibitors, μM (95% CI)						ki-values of covalent inhibitors, μM^-1^ min^-1^ (95% CI)	
Enzyme	Donepezil	C7653	C5685R	C5685S	Ethopropazine	Propoxur	Eserine
***Ag*AChE1**	0.24 (0.19–0.30)	0.44 (0.29–0.67)	>30	>30	8.3 (5.8–11.8)	1.11 (1.02–1.21)	27.3 (23.8–30.8)
***Aa*AChE1**	0.28 (0.18–0.43)	0.36 (0.26–0.49)	>30	>30	4.6 (3.98–5.3)	1.20 (1.05–1.35)	24.8 (18.1–31.5)
***h*AChE**	0.008 (0.0071–0.0085)	0.36	1.3**	1.4**	~1 mM	0.081 (0.071–0.092)	2.66 (2.28–3.05)
***m*AChE**	0.007 (0.0063–0.008)	0.2*	0.7**	0.7**	~1 mM	0.092 (0.068–0.117)	1.36 (1.15–1.56)
***Ag*AChE1-G119S**	0.31 (0.25–0.38)	1.3 (0.8–2)	>30	>30	~1 mM	n. d.	1.18 (0.63–1.72)

*[41]

**[42]

The enzymes were further characterized using a set of non-covalent inhibitors of AChE. The interactions between donepezil, C7653, C5685R, C5685S and vertebrate AChE have been investigated using X-ray crystallography [41, 42, 57], but no crystal structure of AChE in complex with ethopropazine has yet been published. Donepezil and C7653 spans the entire active site gorge, with the benzylic moiety (donepezil) or the piperidine ring (C7653) forming a parallel key interaction with the indole ring of Trp^86^ at the base of the active site gorge [41, 57]. The enantiomeric pair C5685R and C5685S interact with the entrance of the gorge via the substituted phenyl ring, while the N-ethyl pyrrolidine moiety extends towards the catalytic site. Even though the enantiomers are within contact distance of Trp^86^, they do not form close interactions with the indole ring similar to those observed for donepezil and C7653.

We found that C7653 was a potent inhibitor of *Ag*AChE1 and *Aa*AChE1, with *IC*_*50*_-values of 440 and 360 nM respectively, which are similar to those determined for *h*AChE and *m*AChE (Fig 2D and Table 3). Compared to the wild-type enzymes, the G119S mutant slightly reduces the potency of C7653 (*IC*_*50*_ of 1.3 μM). Interestingly, donepezil, C5685R and C5685S all displayed selectivity, favoring the vertebrate form of AChE. For these compounds, the *IC*_*50*_ of *Ag*AChE1-G119S is comparable to the constants determined for *Ag*AChE1 and *Aa*AChE1. This finding indicates that the G119S mutation has only minor implications for the binding of this set of non-covalent ligands. The only exception is ethopropazine, which is not an inhibitor of *m*AChE, *h*AChE or the G119S mutant (*IC*_*50*_ > 1 mM) but does inhibit the wild-type mosquito enzymes with *IC*_*50*_ values of 4.6 and 8.3 μM.

### Cluster analysis of functional descriptors

**Fig 3:**
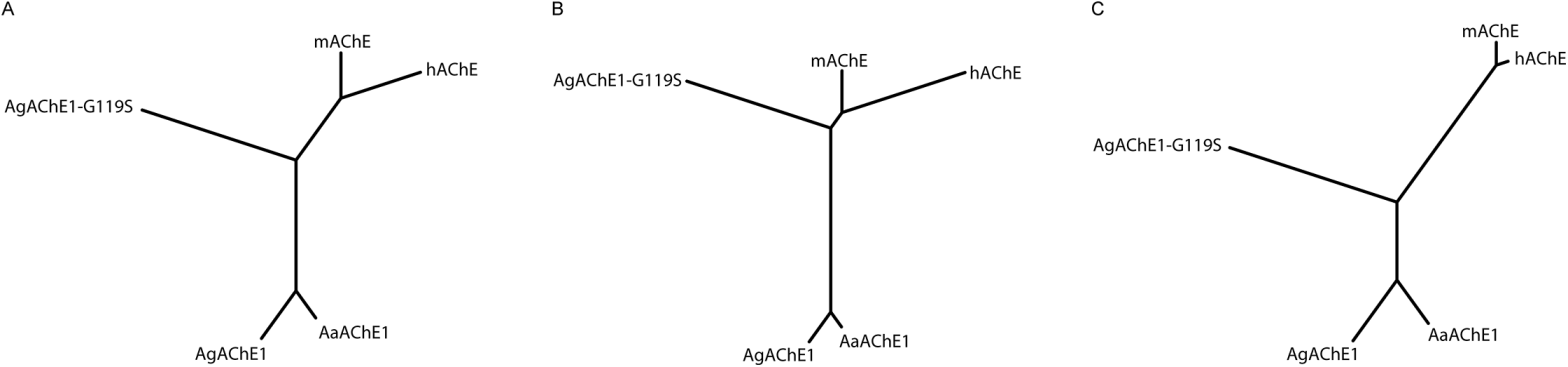
Cluster analysis of functional descriptors. (A) Un-rooted tree based on all experimentally determined parameters (main dataset: *k*_*cat*_, *K*_*M*_, *V*_*max*_, *k*_*i*_ and *IC*_*50*_–values). (B) Un-rooted tree based on parameters related to basal enzymatic function and variables dependent on both affinity and reaction rate (sub-set A: *k*_*cat*_, *K*_*M*_, *V*_*max*_, *k*_*i*_-values). (C) Un-rooted tree based on parameters related to affinity (sub-set B: *IC*_*50*_–values).

A cluster analysis based on the experimentally determined parameters *k*_*cat*_, *K*_*M*_, *V*_*max*_, *k*_*i*_ and the calculated *IC*_*50*_ values (S1 Table) visualized as an un-rooted tree revealed three distinct clusters consisting of the wild-type mosquito enzymes, the *Ag*AChE1-G119S enzyme and finally the vertebrate *m*AChE and *h*AChE (Fig 3A). The data were further divided into two sub-sets. Sub-set A, containing constants that describe the affinity and/or reactivity of compounds that form a covalent bond to the catalytic serine residue, yielded a tree where the two mosquito wild-type enzymes still clustered close together. Interestingly, however, this subset analysis shifted the *Ag*AChE1-G119S branch towards the vertebrate enzymes, indicating a greater degree of similarity in the ligand binding properties (Fig 3B). The cluster analysis based on sub-set B, which contained data for the non-covalent inhibitors (*i*.*e*. compounds that do not form a covalent bond to the catalytic serine residue) yielded a tree where the mosquito wild-type AChE1s and vertebrate proteins again formed two distinct clusters. In contrast to sub-set A, the node of the *Ag*AChE1-G119S branch was shifted from the vertebrate cluster towards the wild-type AChE1 cluster (Fig 3C).

### Summary and conclusions

A critical question for the development of insecticides with improved selectivity is whether AChEs from different species have sufficiently diverse ligand binding properties to allow development of synthetic compounds that are selective towards mosquitoes without affecting the cholinergic transmission in off-target species. Sequence comparisons, crystallographic studies of AChE from various species, and homology modelling of *Ag*AChE1 all suggest that the three dimensional structures of vertebrate and mosquito enzymes are similar [26, 58]. Nevertheless, a free cysteine located in a loop at the entrance of the active gorge, corresponding to Phe^295^ in *h*AChE, structurally diverge from the vertebrate enzymes and is thus a potential target for selective covalent inhibitors [30, 59, 60]. Likewise, recent work targeting the catalytic serine residue with covalent inhibitors containing a carbamate functionality has shown that considerable selectivity ratios can be achieved for mosquito over vertebrate enzymes [31, 32, 54, 61]. While the potential of covalent inhibitors has been investigated in a number of studies, inhibitors with a different mode of action, such as non-covalent inhibitors, are less well explored [62]. Another major concern in insecticide development is to overcome the increasing resistance to AChE inhibitors conferred by *e*.*g*. the G119S mutation. Novel small-core carbamates have been designed [54], and it has also been shown that it is possible to target resistant mutant enzymes with non-covalent inhibitors [62].

In this study, we cloned, expressed and characterized the wild type AChE1 protein from two important disease-vectors and compared these enzymes to orthologous vertebrate AChEs. We found that the mosquito AChE1 enzymes share many functional and structural characteristics with the vertebrate AChE enzymes. For example, analysis of post-translational modifications show that the mosquito enzymes have N-linked glycosylation sites (Table 1). A substrate preference analysis showed that ATChI is the preferred substrate from a set of substrate analogues (Table 2), and the mosquito AChE1 also exhibited a typical substrate inhibition behavior at high substrate concentrations (Fig 2B). To extend the analysis, we generated the insecticide-resistant G119S mutant of *Ag*AChE1 and probed the efficacy and ligand binding properties of the different enzymes with a selection of known substrates and inhibitors. Even though the chemical diversity of the compounds considered in this study is limited, a cluster analysis allowed visualization of functional trends (Fig 3A). As the analysis is based on biochemical constants, the trends are related to the ligand binding properties of the different enzymes. For substrates and covalent inhibitors (*i*.*e*. compounds that react with the catalytic serine residue), we found that the G119S substitution induced significant changes of *Ag*AChE1 and made the ligand binding properties of the mutant protein more similar to the properties of *m*AChE and *h*AChE (Fig 3B). In contrast, visualizing a tree based on the constants determined for the non-covalent inhibitors (*i*.*e*. compounds that do not react with the catalytic serine residue), the G119S substitution instead made the ligand binding properties more similar to the *Ag*AChE1/*Aa*AChE1 wild type proteins (Fig 3C). This finding indicates that within this group of ligands, the non-covalent inhibitors are less sensitive to the G119S resistance mutation. It also indicates that it may be useful to explore compounds that inhibit AChE via a different mode of action than the currently used covalent inhibitors.

## Supporting Information

S1 Fig SDS-PAGE gel showing the molecular weight of purified mature *Ag*AChE1 and *Aa*AChE1 proteins. (DOCX)

S2 Fig Post-translational modifications. (DOCX)

S3 Fig VX-titration curves. (TIF)

S1 Table Kinetic constants included in the cluster analysis of functional descriptors. (DOCX)

S1 Text Additional results and discussion regarding the analysis of post-translational modifications in AChE1 from mosquitoes. (DOCX)

## References

[1] Lindgren E, Andersson Y, Suk JE, Sudre B, Semenza JC. Public health. Monitoring EU emerging infectious disease risk due to climate change. Science (New York, NY). 2012;336(6080):418–9. Epub 2012/04/28. 10.1126/science.1215735 . 22539705

[2] World Malaria Report 20142014.

[3] Bhatt S, Gething PW, Brady OJ, Messina JP, Farlow AW, Moyes CL, et al The global distribution and burden of dengue. Nature. 2013;496(7446):504–7. 10.1038/nature12060 PMC3651993. 23563266 PMC3651993

[4] Sinka ME, Bangs MJ, Manguin S, Rubio-Palis Y, Chareonviriyaphap T, Coetzee M, et al A global map of dominant malaria vectors. Parasites & vectors. 2012;5:69 Epub 2012/04/06. 10.1186/1756-3305-5-69 22475528 PMC3349467

[5] Gubler DJ. The changing epidemiology of yellow fever and dengue, 1900 to 2003: full circle? Comparative immunology, microbiology and infectious diseases. 2004;27(5):319–30. Epub 2004/07/01. 10.1016/j.cimid.2004.03.013 . 15225982

[6] Paupy C, Delatte H, Bagny L, Corbel V, Fontenille D. Aedes albopictus, an arbovirus vector: from the darkness to the light. Microbes and infection / Institut Pasteur. 2009;11(14–15):1177–85. Epub 2009/05/20. 10.1016/j.micinf.2009.05.005 . 19450706

[7] Casida JE, Quistad GB. Golden age of insecticide research: past, present, or future? Annual review of entomology. 1998;43:1–16. Epub 1998/01/28. 10.1146/annurev.ento.43.1.1 . 9444749

[8] Ffrench-Constant RH. The molecular genetics of insecticide resistance. Genetics. 2013;194(4):807–15. Epub 2013/08/03. 10.1534/genetics.112.141895 23908373 PMC3730913

[9] Ranson H, Burhani J, Lumjuan N, Black IV WC. Insecticide resistance in dengue vectors. TropIKAnet. 2010;1:0-.

[10] Rivero A, Vezilier J, Weill M, Read AF, Gandon S. Insecticide control of vector-borne diseases: when is insecticide resistance a problem? PLoS pathogens. 2010;6(8):e1001000 Epub 2010/08/12. 10.1371/journal.ppat.1001000 20700451 PMC2916878

[11] Ranson H, N'Guessan, Lines J, Moiroux N, Nkuni Z, Corbel V. Pyrethroid resistance in African anopheline mosquitoes: what are the implications for malaria control? Trends in parasitology. 2011;27(2):91–8. Epub 2010/09/17. 10.1016/j.pt.2010.08.004 . 20843745

[12] Xi Z, Khoo CC, Dobson SL. Wolbachia establishment and invasion in an Aedes aegypti laboratory population. Science (New York, NY). 2005;310(5746):326–8. Epub 2005/10/15. 10.1126/science.1117607 . 16224027

[13] Bian G, Xu Y, Lu P, Xie Y, Xi Z. The endosymbiotic bacterium Wolbachia induces resistance to dengue virus in Aedes aegypti. PLoS pathogens. 2010;6(4):e1000833 Epub 2010/04/07. 10.1371/journal.ppat.1000833 20368968 PMC2848556

[14] Ledermann JP, Suchman EL, Black WCt, Carlson JO. Infection and pathogenicity of the mosquito densoviruses AeDNV, HeDNV, and APeDNV in Aedes aegypti mosquitoes (Diptera: Culicidae). Journal of economic entomology. 2004;97(6):1828–35. Epub 2005/01/26. . 15666733 10.1093/jee/97.6.1828

[15] Raghavendra K, Barik TK, Reddy BP, Sharma P, Dash AP. Malaria vector control: from past to future. Parasitology research. 2011;108(4):757–79. Epub 2011/01/14. 10.1007/s00436-010-2232-0 . 21229263

[16] Colovic MB, Krstic DZ, Lazarevic-Pasti TD, Bondzic AM, Vasic VM. Acetylcholinesterase inhibitors: pharmacology and toxicology. Current neuropharmacology. 2013;11(3):315–35. Epub 2013/11/02. 10.2174/1570159x11311030006 24179466 PMC3648782

[17] Shafferman A, Kronman C, Flashner Y, Leitner M, Grosfeld H, Ordentlich A, et al Mutagenesis of human acetylcholinesterase. Identification of residues involved in catalytic activity and in polypeptide folding. The Journal of biological chemistry. 1992;267(25):17640–8. Epub 1992/09/05. . 1517212

[18] Sussman JL, Harel M, Frolow F, Oefner C, Goldman A, Toker L, et al Atomic structure of acetylcholinesterase from Torpedo californica: a prototypic acetylcholine-binding protein. Science (New York, NY). 1991;253(5022):872–9. Epub 1991/08/23. . 1678899 10.1126/science.1678899

[19] Hoffmann F, Fournier D, Spierer P. Minigene rescues acetylcholinesterase lethal mutations in Drosophila melanogaster. Journal of molecular biology. 1992;223(1):17–22. Epub 1992/01/05. . 1731068 10.1016/0022-2836(92)90710-2

[20] Weill M, Fort P, Berthomieu A, Dubois MP, Pasteur N, Raymond M. A novel acetylcholinesterase gene in mosquitoes codes for the insecticide target and is non-homologous to the ace gene in Drosophila. Proceedings Biological sciences / The Royal Society. 2002;269(1504):2007–16. Epub 2002/10/25. 10.1098/rspb.2002.2122 12396499 PMC1691131

[21] Kim YH, Lee SH. Which acetylcholinesterase functions as the main catalytic enzyme in the Class Insecta? Insect biochemistry and molecular biology. 2013;43(1):47–53. Epub 2012/11/22. 10.1016/j.ibmb.2012.11.004 . 23168079

[22] Cha DJ, Lee SH. Evolutionary origin and status of two insect acetylcholinesterases and their structural conservation and differentiation. Evolution & development. 2015;17(1):109–19. Epub 2015/01/30. 10.1111/ede.12111 . 25627717

[23] Huchard E, Martinez M, Alout H, Douzery EJ, Lutfalla G, Berthomieu A, et al Acetylcholinesterase genes within the Diptera: takeover and loss in true flies. Proceedings Biological sciences / The Royal Society. 2006;273(1601):2595–604. Epub 2006/09/28. 10.1098/rspb.2006.3621 17002944 PMC1635460

[24] Weill M, Lutfalla G, Mogensen K, Chandre F, Berthomieu A, Berticat C, et al Comparative genomics: Insecticide resistance in mosquito vectors. Nature. 2003;423(6936):136–7. Epub 2003/05/09. 10.1038/423136b . 12736674

[25] Weill M, Berthomieu A, Berticat C, Lutfalla G, Negre V, Pasteur N, et al Insecticide resistance: a silent base prediction. Current biology: CB. 2004;14(14):R552–3. Epub 2004/07/23. 10.1016/j.cub.2004.07.008 . 15268871

[26] Harel M, Kryger G, Rosenberry TL, Mallender WD, Lewis T, Fletcher RJ, et al Three-dimensional structures of Drosophila melanogaster acetylcholinesterase and of its complexes with two potent inhibitors. Protein science: a publication of the Protein Society. 2000;9(6):1063–72. Epub 2000/07/13. 10.1110/ps.9.6.1063 10892800 PMC2144661

[27] Jiang H, Liu S, Zhao P, Pope C. Recombinant expression and biochemical characterization of the catalytic domain of acetylcholinesterase-1 from the African malaria mosquito, Anopheles gambiae. Insect biochemistry and molecular biology. 2009;39(9):646–53. Epub 2009/07/18. 10.1016/j.ibmb.2009.07.002 19607916 PMC2772825

[28] Mori A, Lobo NF, deBruyn B, Severson DW. Molecular cloning and characterization of the complete acetylcholinesterase gene (Ace1) from the mosquito Aedes aegypti with implications for comparative genome analysis. Insect biochemistry and molecular biology. 2007;37(7):667–74. Epub 2007/06/07. 10.1016/j.ibmb.2007.03.014 17550823 PMC2716755

[29] Anthony N, Rocheleau T, Mocelin G, Lee HJ, ffrench-Constant R. Cloning, sequencing and functional expression of an acetylcholinesterase gene from the yellow fever mosquito Aedes aegypti. FEBS letters. 1995;368(3):461–5. Epub 1995/07/24. . 7635199 10.1016/0014-5793(95)00711-h

[30] Pang YP. Novel acetylcholinesterase target site for malaria mosquito control. PloS one. 2006;1:e58 Epub 2006/12/22. 10.1371/journal.pone.0000058 17183688 PMC1762403

[31] Hartsel JA, Wong DM, Mutunga JM, Ma M, Anderson TD, Wysinski A, et al Re-engineering aryl methylcarbamates to confer high selectivity for inhibition of Anopheles gambiae versus human acetylcholinesterase. Bioorganic & medicinal chemistry letters. 2012;22(14):4593–8. Epub 2012/06/29. 10.1016/j.bmcl.2012.05.103 22738634 PMC3389130

[32] Dou D, Park JG, Rana S, Madden BJ, Jiang H, Pang YP. Novel selective and irreversible mosquito acetylcholinesterase inhibitors for controlling malaria and other mosquito-borne diseases. Scientific reports. 2013;3:1068 Epub 2013/01/17. 10.1038/srep01068 23323211 PMC3545233

[33] Mongin E, Louis C, Holt RA, Birney E, Collins FH. The Anopheles gambiae genome: an update. Trends in parasitology. 2004;20(2):49–52. Epub 2004/01/30. . 14747013 10.1016/j.pt.2003.11.003

[34] Nakamura Y, Gojobori T, Ikemura T. Codon usage tabulated from international DNA sequence databases: status for the year 2000. Nucleic acids research. 2000;28(1):292 10592250 10.1093/nar/28.1.292 PMC102460

[35] Hopkins R, Esposito D. A rapid method for titrating baculovirus stocks using the Sf-9 Easy Titer cell line. BioTechniques. 2009;47(3):785–8. 10.2144/000113238 . 19852765

[36] LaBarre DD, Lowy RJ. Improvements in methods for calculating virus titer estimates from TCID50 and plaque assays. Journal of virological methods. 2001;96(2):107–26. Epub 2001/07/11. . 11445142 10.1016/s0166-0934(01)00316-0

[37] Artursson E, Akfur C, Hornberg A, Worek F, Ekstrom F. Reactivation of tabun-hAChE investigated by structurally analogous oximes and mutagenesis. Toxicology. 2009;265(3):108–14. Epub 2009/09/19. 10.1016/j.tox.2009.09.002 . 19761810

[38] Ekstrom F, Akfur C, Tunemalm AK, Lundberg S. Structural changes of phenylalanine 338 and histidine 447 revealed by the crystal structures of tabun-inhibited murine acetylcholinesterase. Biochemistry. 2006;45(1):74–81. Epub 2006/01/04. 10.1021/bi051286t . 16388582

[39] Fredriksson SA, Hulst AG, Artursson E, de Jong AL, Nilsson C, van Baar BL. Forensic identification of neat ricin and of ricin from crude castor bean extracts by mass spectrometry. Analytical chemistry. 2005;77(6):1545–55. Epub 2005/03/15. 10.1021/ac048756u . 15762556

[40] Ellman GL, Courtney KD, Andres V Jr., Feather-Stone RM. A new and rapid colorimetric determination of acetylcholinesterase activity. Biochemical pharmacology. 1961;7:88–95. . 13726518 10.1016/0006-2952(61)90145-9

[41] Berg L, Andersson CD, Artursson E, Hornberg A, Tunemalm AK, Linusson A, et al Targeting acetylcholinesterase: identification of chemical leads by high throughput screening, structure determination and molecular modeling. PloS one. 2011;6(11):e26039 Epub 2011/12/06. 10.1371/journal.pone.0026039 22140425 PMC3227566

[42] Berg L, Niemiec MS, Qian W, Andersson CD, Wittung-Stafshede P, Ekstrom F, et al Similar but different: thermodynamic and structural characterization of a pair of enantiomers binding to acetylcholinesterase. Angewandte Chemie (International ed in English). 2012;51(51):12716–20. Epub 2012/11/20. 10.1002/anie.201205113 . 23161758

[43] Sugimoto H, Yamanishi Y, Iimura Y, Kawakami Y. Donepezil hydrochloride (E2020) and other acetylcholinesterase inhibitors. Current medicinal chemistry. 2000;7(3):303–39. Epub 2000/01/19. . 10637367 10.2174/0929867003375191

[44] Saitou N, Nei M. The neighbor-joining method: a new method for reconstructing phylogenetic trees. Molecular biology and evolution. 1987;4(4):406–25. Epub 1987/07/01. . 3447015 10.1093/oxfordjournals.molbev.a040454

[45] Felsenstein J. PHYLIP (Phylogeny Inference Package) version 3.6 3.68 ed. Department of Genome Sciences, University of Washington, Seattle, http://evolution.genetics.washington.edu/phylip.html: Distributed by the author; 2005.

[46] Bateman KP, White R.L., Yaguchi M., Thibault P. Characterization of protein glycoforms by capillary-zone electrophoresis–nanoelectrospray mass spectrometry. Journal of Chromatography. 1998;794:327–44.

[47] Saxena A, Raveh L, Ashani Y, Doctor BP. Structure of glycan moieties responsible for the extended circulatory life time of fetal bovine serum acetylcholinesterase and equine serum butyrylcholinesterase. Biochemistry. 1997;36(24):7481–9. Epub 1997/06/17. 10.1021/bi963156d . 9200697

[48] Ordentlich A, Barak D, Kronman C, Flashner Y, Leitner M, Segall Y, et al Dissection of the human acetylcholinesterase active center determinants of substrate specificity. Identification of residues constituting the anionic site, the hydrophobic site, and the acyl pocket. The Journal of biological chemistry. 1993;268(23):17083–95. Epub 1993/08/15. . 8349597

[49] Augustinsson KB, Nachmansohn D. Distinction between Acetylcholine-Esterase and Other Choline Ester-splitting Enzymes. Science (New York, NY). 1949;110(2847):98–9. Epub 1949/07/22. 10.1126/science.110.2847.98 . 17837670

[50] Vellom DC, Radic Z, Li Y, Pickering NA, Camp S, Taylor P. Amino acid residues controlling acetylcholinesterase and butyrylcholinesterase specificity. Biochemistry. 1993;32(1):12–7. Epub 1993/01/12. . 8418833 10.1021/bi00052a003

[51] Hsiao YM, Lai JY, Liao HY, Feng HT. Purification and characterization of acetylcholinesterase from oriental fruit fly [Bactrocera dorsalis (Hendel)] (Diptera: Tephritidae). Journal of agricultural and food chemistry. 2004;52(17):5340–6. Epub 2004/08/19. 10.1021/jf0494377 . 15315367

[52] Temeyer KB, Brake DK, Tuckow AP, Li AY, Perez de Leon AA. Acetylcholinesterase of the sand fly, Phlebotomus papatasi (Scopoli): cDNA sequence, baculovirus expression, and biochemical properties. Parasites & vectors. 2013;6:31 Epub 2013/02/06. 10.1186/1756-3305-6-31 23379291 PMC3598880

[53] Radic Z, Pickering NA, Vellom DC, Camp S, Taylor P. Three distinct domains in the cholinesterase molecule confer selectivity for acetyl- and butyrylcholinesterase inhibitors. Biochemistry. 1993;32(45):12074–84. Epub 1993/11/16. . 8218285 10.1021/bi00096a018

[54] Wong DM, Li J, Chen QH, Han Q, Mutunga JM, Wysinski A, et al Select small core structure carbamates exhibit high contact toxicity to "carbamate-resistant" strain malaria mosquitoes, Anopheles gambiae (Akron). PloS one. 2012;7(10):e46712 Epub 2012/10/11. 10.1371/journal.pone.0046712 23049714 PMC3462181

[55] Organization WH. WHO Pesticide Evaluation Scheme (WHOPES): WHO specifications for pesticides used in public health: Propoxur: WHO; 2015 [cited 2015 12th May].

[56] Saxena A, Redman AM, Jiang X, Lockridge O, Doctor BP. Differences in active-site gorge dimensions of cholinesterases revealed by binding of inhibitors to human butyrylcholinesterase. Chemico-biological interactions. 1999;119–120:61–9. Epub 1999/07/27. . 10421439 10.1016/s0009-2797(99)00014-9

[57] Kryger G, Silman I, Sussman JL. Three-dimensional structure of a complex of E2020 with acetylcholinesterase from Torpedo californica. Journal of physiology, Paris. 1998;92(3–4):191–4. Epub 1998/10/28. . 9789806 10.1016/s0928-4257(98)80008-9

[58] Pang YP, Brimijoin S, Ragsdale DW, Zhu KY, Suranyi R. Novel and viable acetylcholinesterase target site for developing effective and environmentally safe insecticides. Current drug targets. 2012;13(4):471–82. Epub 2012/01/28. 22280344 10.2174/138945012799499703 PMC3343382

[59] Pang YP. Species marker for developing novel and safe pesticides. Bioorganic & medicinal chemistry letters. 2007;17(1):197–9. Epub 2006/10/19. 10.1016/j.bmcl.2006.09.073 . 17046256

[60] Pezzementi L, Rowland M, Wolfe M, Tsigelny I. Inactivation of an invertebrate acetylcholinesterase by sulfhydryl reagents: the roles of two cysteines in the catalytic gorge of the enzyme. Invertebrate neuroscience: IN. 2006;6(2):47–55. Epub 2006/04/06. 10.1007/s10158-006-0017-z . 16586114

[61] Jiang Y, Swale D, Carlier PR, Hartsel JA, Ma M, Ekström F, et al Evaluation of novel carbamate insecticides for neurotoxicity to non-target species. Pesticide Biochemistry and Physiology. 2013;106(3):156–61. doi: 10.1016/j.pestbp.2013.03.006.

[62] Alout H, Labbe P, Berthomieu A, Djogbenou L, Leonetti JP, Fort P, et al Novel AChE inhibitors for sustainable insecticide resistance management. PloS one. 2012;7(10):e47125 Epub 2012/10/12. 10.1371/journal.pone.0047125 23056599 PMC3466212

