# Supplementary material for: Acetylcholinesterases from the Disease Vectors Aedes aegypti and Anopheles gambiae: Functional Characterization and Comparisons with Vertebrate Orthologues
Source: PLoS One. 2015 Oct 8;10(10):e0138598. doi: 10.1371/journal.pone.0138598 (PMC4598118; doi:10.1371/journal.pone.0138598)

### S1 Figure – SDS-PAGE gel showing the molecular weight of purified mature *Ag*AChE1 and *Aa*AChE1 proteins.


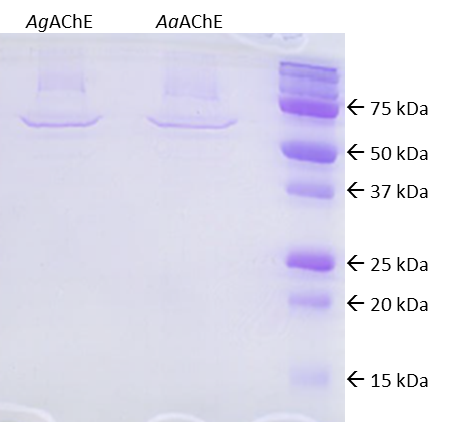

Supplement: S1 Fig — (DOCX) [file pone.0138598.s001.docx]
