# Supplementary material for: Acetylcholinesterases from the Disease Vectors Aedes aegypti and Anopheles gambiae: Functional Characterization and Comparisons with Vertebrate Orthologues
Source: PLoS One. 2015 Oct 8;10(10):e0138598. doi: 10.1371/journal.pone.0138598 (PMC4598118; doi:10.1371/journal.pone.0138598)
Supplement: S2 Fig — (DOCX) [file pone.0138598.s002.docx]

**S2 Figure - Post-translational modifications.**


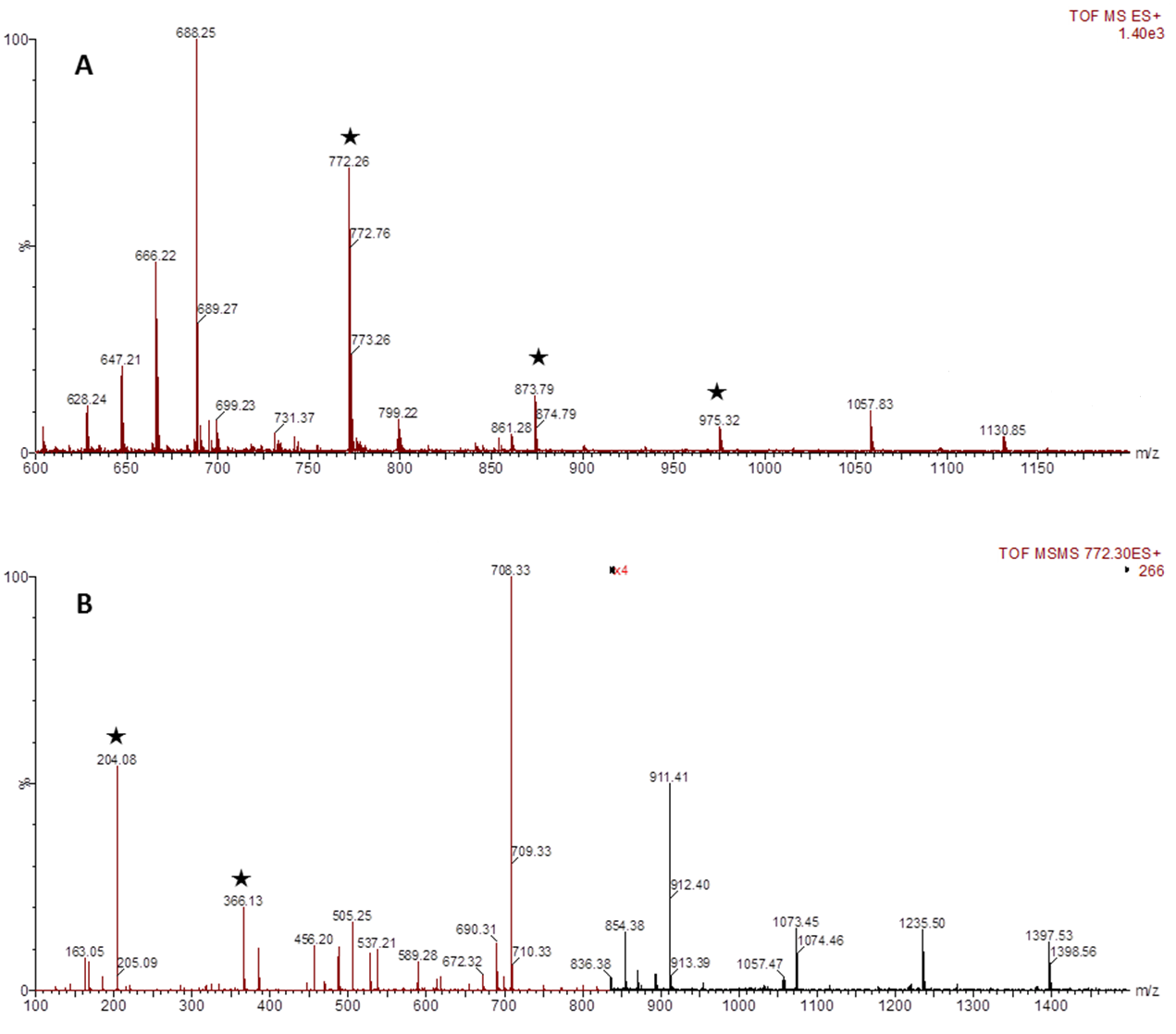


**(A)** Low energy LC-MS spectrum of glycopeptides in the *Aa*AChE1*-*digest indicated by carbohydrate marker ions. Doubly charged glycopeptide candidate ions at *m/z* 772.27^2+^, 873.79^2+^, 975.32^2+^ (★).

**(B)** Product ion spectrum of *m/z* 772.27^2+^. Glycopeptide marker ions are found at 204 and 366. Glycan sequence ions at 1397.53, 1235.50, 1073.45, 911.41 and 708.33 are consistent with a fucosylated N-glycan core structure GlcNAc(Fuc)-GlcNAc-Man_3_. The ion at 854.38 locates the fucose on the N-acetylglucosamine attached to the peptide backbone.
