# Supplementary material for: Acetylcholinesterases from the Disease Vectors Aedes aegypti and Anopheles gambiae: Functional Characterization and Comparisons with Vertebrate Orthologues
Source: PLoS One. 2015 Oct 8;10(10):e0138598. doi: 10.1371/journal.pone.0138598 (PMC4598118; doi:10.1371/journal.pone.0138598)
Supplement: S1 Table — (DOCX) [file pone.0138598.s004.docx]

**S1 Table – Kinetic constants included in the cluster analysis of functional descriptors.**

|  |  | ***Aa*AChE1** | ***Ag*AChE1** | ***Ag*AChE1-G119S** | ***m*AChE** | ***h*AChE** |
| --- | --- | --- | --- | --- | --- | --- |
| ***V_max_* (mA/min)** | ATChI | 100^a^ | 100^a^ | 100^a^ | 100^a^ | 100^a^ |
|  | PTChI | 65,5^a^ | 77,1^a^ | 26,4^a^ | 50,6^a^ | 52,3^a^ |
| ***K_M_* (µM)** | ATChI | 25 | 27 | 58 | 84 | 146 |
|  | PTChI | 20 | 25 | 315 | 59 | 150 |
| ***K_cat_* (s^-1^)** | ATChI | 140 | 124 | 4^b^ | 2333^c^ | 6167^d^ |
| ***k_i_* (µM^-1^ min^-1^)** | eserine | 24,8 | 27,26 | 1,18 | 1,36 | 2,66 |
|  | propoxur | 1,2 | 1,11 | 0,00015^b^ | 0,092 | 0,081 |
| ***IC_50_* (µM)** | ethopropazine | 4,6 | 8,3 | 1000 | 1000 | 1000 |
|  | donepezil | 0,28 | 0,24 | 0,31 | 0,007 | 0,008 |
|  | C7653 | 0,36 | 0,44 | 1,3 | 0,2^e^ | 0,36 |
|  | C5685R | 21 | 58 | 69 | 0,7^f^ | 1,3^f^ |
|  | C5685S | 210 | 285 | 63 | 0,7^f^ | 1,4^f^ |

^a^In percent of V_max_(ATChI) ^b^Estimated values based on [[54](#_ENREF_54)], ^c^[[53](#_ENREF_53)] ^d^[[48](#_ENREF_48)] ^e^[[41](#_ENREF_41)] ^f^[[42](#_ENREF_42)]
