# Supplementary material for: Acetylcholinesterases from the Disease Vectors Aedes aegypti and Anopheles gambiae: Functional Characterization and Comparisons with Vertebrate Orthologues
Source: PLoS One. 2015 Oct 8;10(10):e0138598. doi: 10.1371/journal.pone.0138598 (PMC4598118; doi:10.1371/journal.pone.0138598)
Supplement: S1 Text — (DOCX) [file pone.0138598.s005.docx]

**S1 Text**

Figure S2A shows the low energy spectrum in the retention time window where carbohydrate marker ions indicated the presence of glycopeptides. Ions 204 and 366 in the product ion spectrum of 772.26 confirmed that this was a glycopeptide molecular ion. The monosaccharide composition of the glycan was obtained from the glycan sequence ions at higher mass (Figure S2B). The ion at 1397 corresponds to a loss of fucose (146) from the molecular ion followed by loss of three mannoses indicated by ions separated by 162 down to *m/z* 911. The loss of 203 from the ion at 911 to give 708 corresponds to the second glucosamine. The ion at 854.38 locates the fucose on the GlcNAc attached to the peptide backbone.

The fragment ion at m/z 708.33 is a characteristic ion (the Y1 ion) in the CID spectrum of glycopeptides with N-linked structures [46] and emanates from cleavage between the two glucosamines (GlcNAc) attached to the peptide backbone. From this ion the mass of both the unsubstituted peptide and the intact glycan was calculated (Table 1), where the glycan mass matched that of ‑GlcNAc(Fuc)-GlcNAc-Man_3_, *i.e.* the core structure of N-linked oligosaccharides (theor. 1038.38).

To identify the specific glycosylation site the peptide mass was matched to the amino acid sequence of the protein. Among the six amino acid sequences in the *Ae. aegypti* digest that matched the mass within ±0.10, the pentapeptide GLNTT with a mass of 504.25 (Gly507 to Thr511, hAChE numbering) was the only sequence that contained the N-glycosylation consensus sequence (Asn-Xxx-Ser/Thr). For *An. gambiae* the GLNTS sequence (Gly507 to Ser511, hAChE numbering) was the only sequence out of ten with a mass within 0.04 that could be N-glycosylated.

In the *Ae. aegypti* digest all glycopeptides in the same retention time window had a Y1 ion at *m/z* 708.33 and are thus different glycoforms of the same peptide (data not shown). The corresponding data was obtained for *An. gambiae.* Table 1 shows the mass of the glycopeptides detected in *Ae. aegypti* and *An. gambiae* AChE1 and the corresponding glycan composition and amino acid sequences. The glycan structures are consistent with the biantennary complex type structures reported earlier in fetal bovine serum AChE and equine serum BuChE [[62](#_ENREF_62)].
